# Publisher Correction: Dimethyl fumarate and 4-octyl itaconate are anticoagulants that suppress Tissue Factor in macrophages via inhibition of Type I Interferon

**DOI:** 10.1038/s41467-023-40034-1

**Published:** 2023-07-20

**Authors:** Tristram A. J. Ryan, Alexander Hooftman, Aisling M. Rehill, Matt D. Johansen, Eóin C. O’ Brien, Juliana E. Toller-Kawahisa, Mieszko M. Wilk, Emily A. Day, Hauke J. Weiss, Pourya Sarvari, Emilio G. Vozza, Fabian Schramm, Christian G. Peace, Alessia Zotta, Stefan Miemczyk, Christina Nalkurthi, Nicole G. Hansbro, Gavin McManus, Laura O’Doherty, Siobhan Gargan, Aideen Long, Jean Dunne, Clíona Ní Cheallaigh, Niall Conlon, Michael Carty, Padraic G. Fallon, Kingston H. G. Mills, Emma M. Creagh, James S. O’ Donnell, Paul J. Hertzog, Philip M. Hansbro, Rachel M. McLoughlin, Małgorzata Wygrecka, Roger J. S. Preston, Zbigniew Zasłona, Luke A. J. O’Neill

**Affiliations:** 1 School of Biochemistry and Immunology, Trinity Biomedical Sciences Institute, Trinity College Dublin, Dublin 2, Ireland; 2 Irish Centre for Vascular Biology, School of Pharmacy and Biomolecular Sciences, RCSI University of Medicine and Health Sciences, Dublin 2, Ireland; 3 Centre for Inflammation, Centenary Institute and University of Technology Sydney, Faculty of Science, Sydney, NSW Australia; 4 Host Pathogen Interactions Group, School of Biochemistry and Immunology, Trinity Biomedical Sciences Institute, Trinity College Dublin, Dublin 2, Ireland; 5 Department of Immunology, Faculty of Biochemistry, Biophysics and Biotechnology, Jagiellonian University, Kraków, Poland; 6 Center for Infection and Genomics of the Lung, German Center for Lung Research (DZL), Faculty of Medicine, Justus Liebig University, Giessen, Germany; 7 Department of Infectious Diseases, St. James’s Hospital, Dublin, Ireland; 8 Clinical Research Facility, St. James’s Hospital, Dublin, Ireland; 9 Department of Clinical Medicine, School of Medicine, Trinity Translational Medicine Institute, Trinity College Dublin, Dublin, Ireland; 10 Department of Immunology, St James’s Hospital, Dublin, Ireland; 11 School of Medicine, Trinity Biomedical Sciences Institute, Trinity College, Dublin 2, Ireland; 12 Centre for Innate Immunity and Infectious Diseases, Hudson Institute of Medical Research, Clayton, VIC Australia; 13 Department of Molecular and Translational Science, Monash University, Clayton, VIC Australia

**Keywords:** Phagocytes, Interferons, Coagulation system, Inflammasome, Sepsis

Correction to: *Nature Communications* 10.1038/s41467-023-39174-1, published online 14 June 2023

The original version of this Article contained an error in the spelling of the surname for the author Luke A J O’Neill, which was incorrectly given as ‘O’ Neill’ with an additional space. This has now been corrected in both the PDF and HTML versions of the Article.

