# Supplementary material for: Dimethyl fumarate and 4-octyl itaconate are anticoagulants that suppress Tissue Factor in macrophages via inhibition of Type I Interferon
Source: Nat Commun. 2023 Jun 14;14:3513. doi: 10.1038/s41467-023-39174-1 (PMC10265568; doi:10.1038/s41467-023-39174-1)

Supplementary Information for:

Dimethyl fumarate and 4-octyl itaconate are anticoagulants that suppress Tissue Factor in macrophages via inhibition of Type I Interferon

Tristram A.J. Ryan[1], Alexander Hooftman[1], Aisling M. Rehill[2], Matt D. Johansen[3], Eóin C. O'Brien[4], Juliana E. Toller-Kawahisa[1], Mieszko M. Wilk[1,5], Emily A. Day[1], Hauke J. Weiss[1], Pourya Sarvari[6], Emilio G. Vozza[4], Fabian Schramm[6], Christian G. Peace[1], Alessia Zotta[1], Stefan Miemczyk[3], Christina Nalkurthi[3], Nicole G. Hansbro[3], Gavin McManus[1], Laura O'Doherty[7,8,9], Siobhan Gargan[9], Aideen Long[9], Jean Dunne[10], Clíona Ní Cheallaigh[7,9], Niall Conlon[8,9,10], Michael Carty[1], Padraic G. Fallon[9,11], Kingston H. G. Mills[1], Emma M. Creagh[1], James S. O'Donnell[2], Paul J. Hertzog[12,13], Philip M. Hansbro[3], Rachel M. McLoughlin[4], Małgorzata Wygrecka[6], Roger J.S. Preston[2], Zbigniew Zasłona[1], Luke A.J. O'Neill[1,14]

[1] School of Biochemistry and Immunology, Trinity Biomedical Sciences Institute, Trinity College Dublin, Dublin 2, Ireland

[2] Irish Centre for Vascular Biology, School of Pharmacy and Biomolecular Sciences, RCSI University of Medicine and Health Sciences, Dublin 2, Ireland

[3] Centre for Inflammation, Centenary Institute and University of Technology Sydney, Faculty of Science, Sydney, NSW, Australia

[4] Host Pathogen Interactions Group, School of Biochemistry and Immunology, Trinity Biomedical Sciences Institute, Trinity College Dublin, Dublin 2, Ireland

[5] Department of Immunology, Faculty of Biochemistry, Biophysics and Biotechnology, Jagiellonian University, Kraków, Poland

[6] Center for Infection and Genomics of the Lung, German Center for Lung Research (DZL), Faculty of Medicine, Justus Liebig University, Giessen, Germany

[7] Department of Infectious Diseases, St. James's Hospital, Dublin, Ireland

[8] Clinical Research Facility, St. James's Hospital, Dublin, Ireland

[9] Department of Clinical Medicine, School of Medicine, Trinity Translational Medicine Institute, Trinity College Dublin, Dublin, Ireland

[10] Department of Immunology, St James's Hospital, Dublin, Ireland

[11] School of Medicine, Trinity Biomedical Sciences Institute, Trinity College, Dublin 2, Ireland

[12] Centre for Innate Immunity and Infectious Diseases, Hudson Institute of Medical Research, Clayton, VIC, Australia
[13] Department of Molecular and Translational Science, Monash University, Clayton, VIC, Australia
[14] Lead contact & correspondence: laoneill@tcd.ie

Table of Contents

Supplementary Figures and Legends	Pages 4-12
Supplementary Table	Page 13
Source Data for Supplementary Figure 3a	Page 14

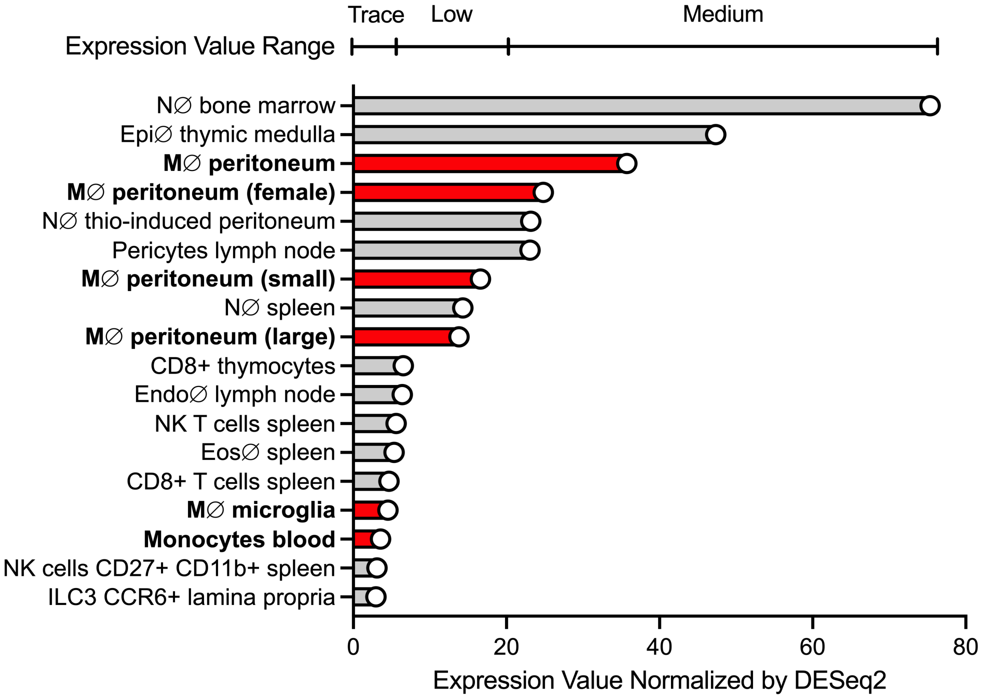

Supplementary Figure 1 – Macrophages are a major expressor of *F3* mRNA

F3 mRNA expression analysis by cell type from the Immunological Genome Project (ImmGen) bulk-population RNA-seq database[38]. Source data are provided as a Source Data file.

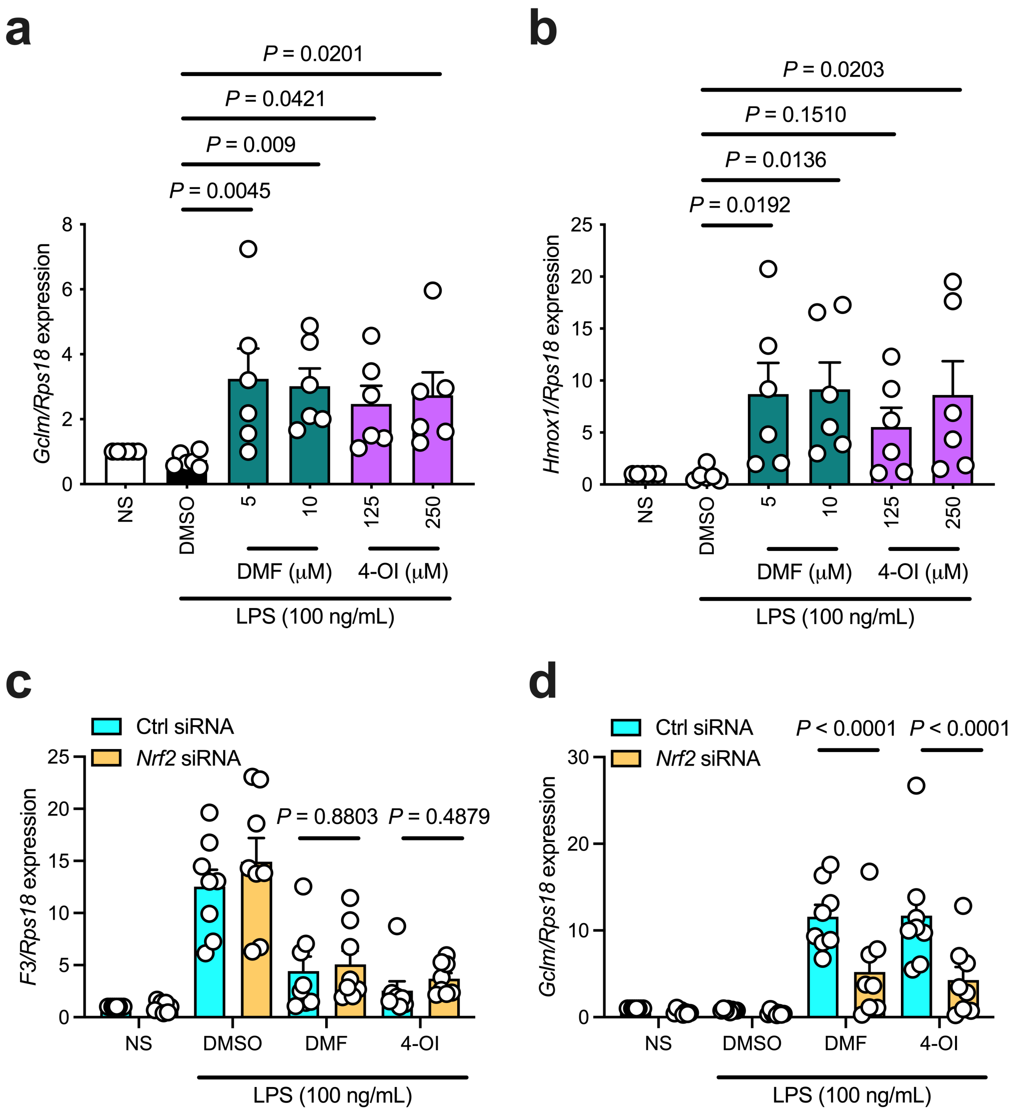

Supplementary Figure 2 – Inhibition of LPS-induced *F3* expression by DMF and 4-OI is independent of NRF2 activation

(a-b) BMDMs were pre-treated with DMSO, DMF, or 4-OI (1 h) before LPS priming (3 h) and harvesting cell lysates. **(a)** *Gclm* and **(b)** *Hmox1* mRNA were quantified by qRT-PCR. **(c-d)** BMDMs were transfected with Ctrl or *Nrf2* siRNA, before treatment with DMSO, DMF, or 4-OI (1 h) prior to LPS stimulation (3 h). **(c)** *F3* and **(d)** *Gclm* mRNA were quantified by qRT-PCR. **(d)** Ctrl siRNA DMF – *Nrf2* siRNA DMF, P = 0.00000976; Ctrl siRNA 4-OI – *Nrf2* siRNA 4-OI, P = 0.0000007. Data from **(a-d)** are mean ± SEM from 3 independent experiments. P values calculated using **(a-b)** one-way ANOVA and **(c-d)** two-way ANOVA for multiple comparisons. Source data are provided as a Source Data file.

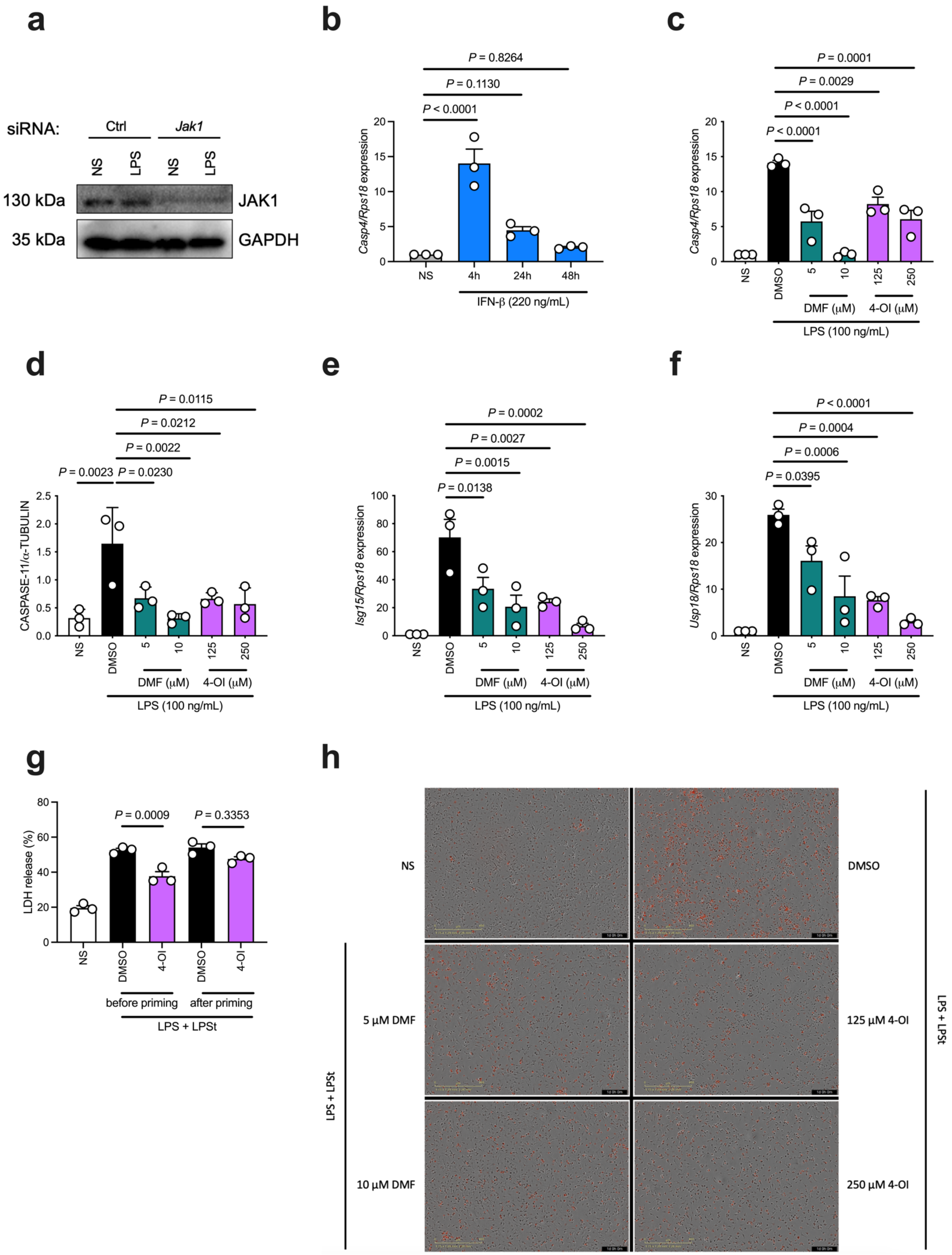
a
siRNA:
Ctrl
Jak1
NS
LPS
NS
LPS
130 kDa
JAK1
35 kDa
GAPDH
b
P = 0.8264
P = 0.1130
P < 0.0001
Casp4/Rps18 expression
NS
4h
24h
48h
IFN-β (220 ng/mL)
c
P = 0.0001
P = 0.0029
P < 0.0001
P < 0.0001
Casp4/Rps18 expression
NS
DMSO
5
10
125
250
DMF (μM)
4-OI (μM)
LPS (100 ng/mL)
d
P = 0.0115
P = 0.0212
P = 0.0022
P = 0.0023
P = 0.0230
CASPASE-11/α-TUBULIN
NS
DMSO
5
10
125
250
DMF (μM)
4-OI (μM)
LPS (100 ng/mL)
e
P = 0.0002
P = 0.0027
P = 0.0015
P = 0.0138
Isg15/Rps18 expression
NS
DMSO
5
10
125
250
DMF (μM)
4-OI (μM)
LPS (100 ng/mL)
f
P < 0.0001
P = 0.0004
P = 0.0006
P = 0.0395
Usp18/Rps18 expression
NS
DMSO
5
10
125
250
DMF (μM)
4-OI (μM)
LPS (100 ng/mL)
g
P = 0.0009
P = 0.3353
LDH release (%)
NS
DMSO
4-OI
DMSO
4-OI
before priming
after priming
LPS + LPSt
h
NS
DMSO
5 μM DMF
125 μM 4-OI
10 μM DMF
250 μM 4-OI
LPS + LPSt
LPS + LPSt

Supplementary Figure 3 – DMF and 4-OI inhibit type I IFN- and caspase-11-mediated pyroptosis in macrophages

(a) Representative western blot of JAK1 in BMDM cell lysates following transfection with Ctrl or *Jak1* siRNA prior to stimulation with LPS (3 h). GAPDH is used as loading control. Blot is representative of 3 independent experiments. **(b)** BMDMs were stimulated with recombinant mouse IFN-β for a timecourse as indicated. *Casp4* (caspase-11) mRNA was quantified by qRT-PCR. NS – 4h, *P* = 0.00002. **(c)** BMDMs were pre-treated DMSO, DMF, or 4-OI (1 h) before LPS priming (3 h). *Casp4* (caspase-11) mRNA was quantified by qRT-PCR. DMSO – 5 μM DMF, *P* = 0.0000868; DMSO – 10 μM DMF, *P* = 0.0000004. **(d)** Densitometry analysis of CASPASE-11 in BMDM cell lysates pre-treated with DMSO, DMF, or 4-OI (1 h) prior to LPS stimulation (3 h), with α-TUBULIN as loading control. **(e-f)** BMDMs were pre-treated with DMSO, DMF, or 4-OI (1 h) before LPS priming (3 h). **(e)** *Isg15* and **(f)** *Usp18* mRNA (DMSO – 250 μM 4-OI, *P* = 0.00004) were quantified by qRT-PCR. **(g)** BMDMs were either pre-treated or post-treated as indicated with DMSO or 4-OI (1 h) before or after priming with LPS (3 h) followed by LPS transfection (16 h). Pyroptosis is represented as percentage cell death measured by LDH release in BMDM supernatants. **(h)** BMDMs were pre-treated with DMSO, DMF, or 4-OI (1 h) before priming with LPS (3 h) and LPS transfection (16 h). Representative images of pyroptotic cell death after propidium iodide staining was added to cell media at time of LPS transfection. Red hue indicates pyroptotic cell death. Scale bar = 400 μm. Images are representative of 3 independent experiments. Data from **(b-c** and **e-g)** are mean ± SEM from 3 independent experiments. Data from **(d)** are mean ± SD from 3 independent experiments. *P* values calculated using two-tailed Student's t-test for paired comparisons or one-way ANOVA for multiple comparisons. Source data are provided as a Source Data file.

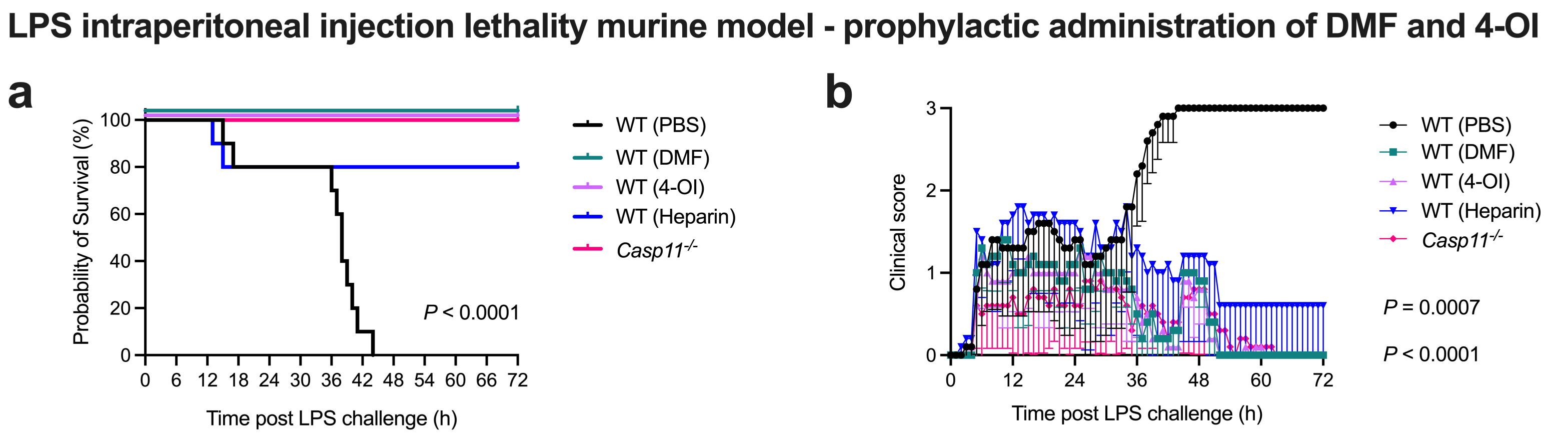

Supplementary Figure 4 – Prophylactic administration of DMF and 4-OI improves survival *in vivo*

(a) Kaplan-Meier survival curve of mice pre-treated with PBS, 50 mg/kg DMF, or 50 mg/kg 4-OI (1 h), or 200 IU/kg heparin (30 min), followed by intraperitoneal injection with 15 mg/kg LPS (for a further 72 h) (n = 10 per group). PBS+LPS – treatment groups, $P = 0.0000000001$. **(b)** Mice from **(a)** were scored clinically (assessing weight loss, activity level, eye closure, and appearance of fur and posture) every hour for 72 hours. PBS+LPS – DMF+LPS, $P = 0.00000000019$. Data points indicate individual mice in **(a-b)**. For **(a-b)** Mantel-Cox survival analysis was performed. Data from **(a-b)** are mean ± SD. Source data are provided as a Source Data file.

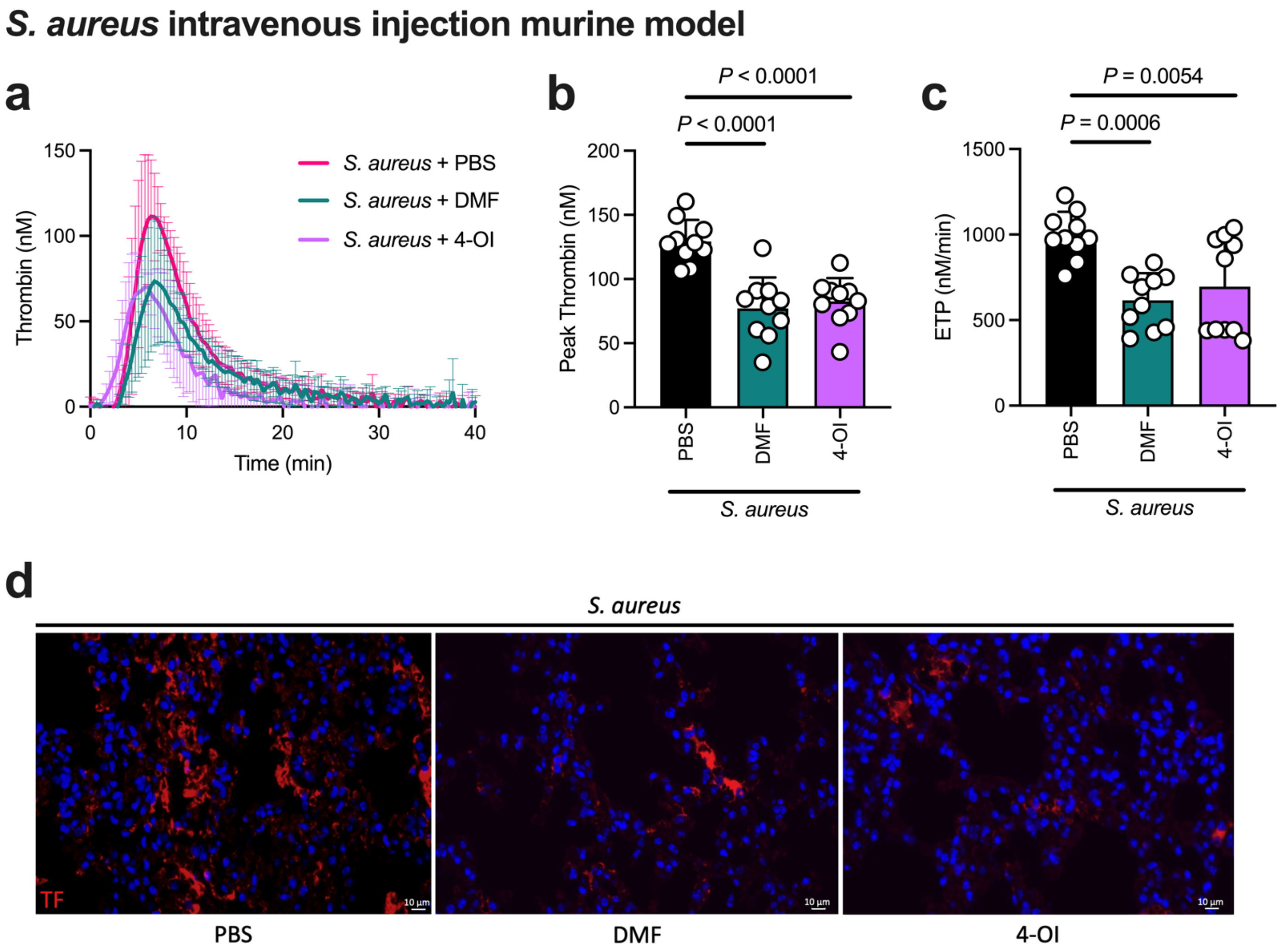

Supplementary Figure 5 – DMF and 4-OI suppress thrombin generation *in vivo* after infection with *S. aureus*

(a) Mice were intravenously injected with 5x10^{7} CFU *S. aureus* (USA300-LAC) and co-treated with PBS, 50 mg/kg DMF, or 50 mg/kg 4-OI (6 h). Citrated plasma was harvested and thrombin generation was assessed (n = 10 per group). **(b)** Peak thrombin generation in mouse citrated plasma treated as in **(a)**. PBS – DMF, *P* = 0.0000067; PBS – 4-OI, *P* = 0.00003. **(c)** Total thrombin generation in mouse citrated plasma treated as in **(a)**. **(d)** TF-positive regions (red) in the lungs of mice treated as in **(a)**. Representative lung tissue sections are shown. Magnification 20×. Scale bar = 10 µm. Data from **(a-c)** are mean ± SD. *P* values calculated using one-way ANOVA for multiple comparisons. Source data are provided as a Source Data file.

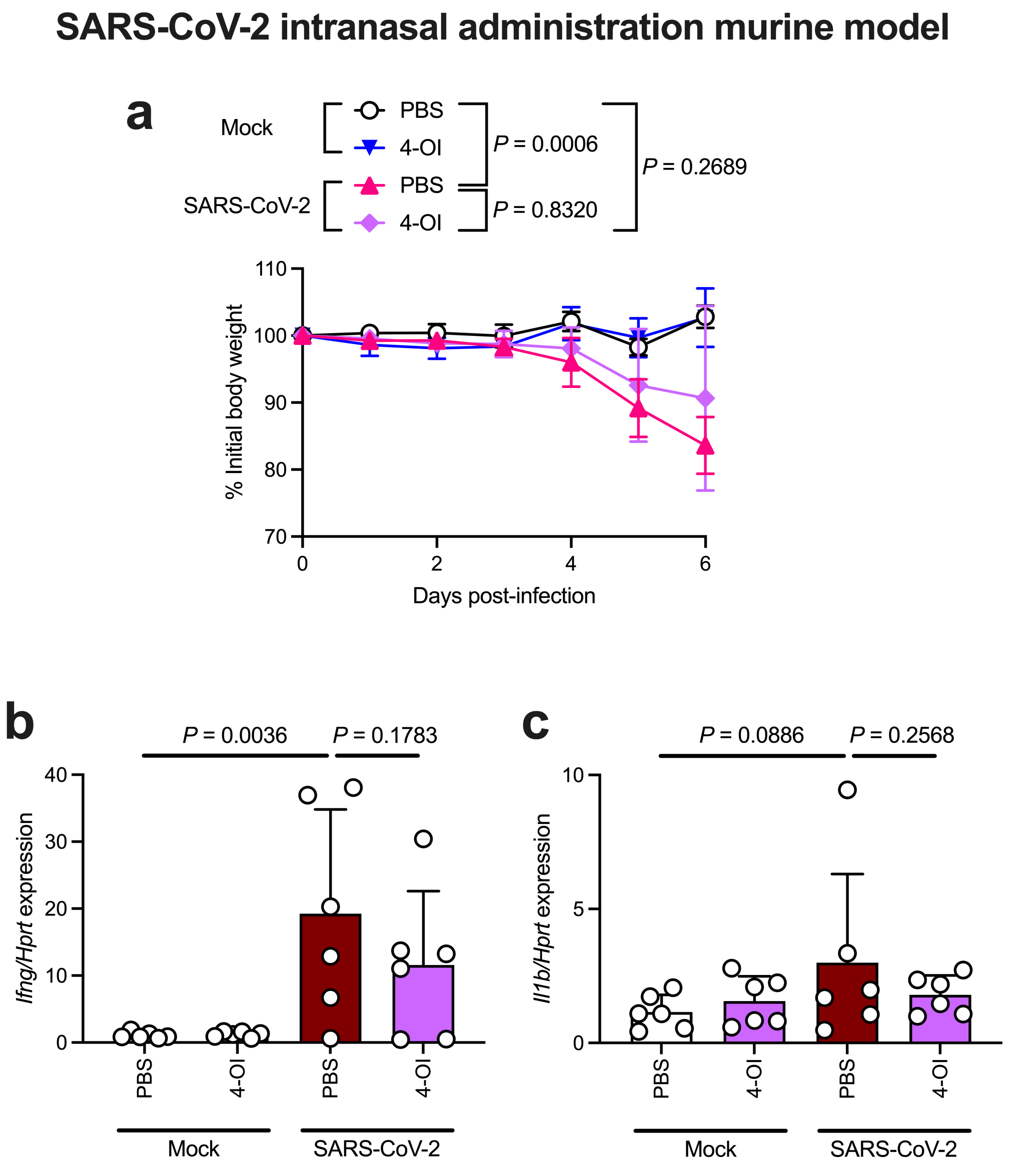

Supplementary Figure 6 – 4-OI limits weight loss and proinflammatory gene induction *in vivo* after SARS-CoV-2 infection

(a) Daily body weight measurements from male K18-hACE2 mice infected with 10^3 PFU SARS-CoV-2 (Wuhan isolate; VIC01/2020) or mock infection and intranasally treated with PBS or 4-OI (10 mg/kg) from 1 day post-infection, with daily treatments continuing until the termination of the experiment at day 6 post-infection (n = 5-6 per group). Quantification of **(b)** *Ifng* and **(c)** *Il1b* mRNA by qRT-PCR in PBMCs of mice treated as in **(a)** (n = 5-6 per group). Data from **(a-c)** are mean ± SD. *P* values calculated using one-way ANOVA for multiple comparisons. Source data are provided as a Source Data file.

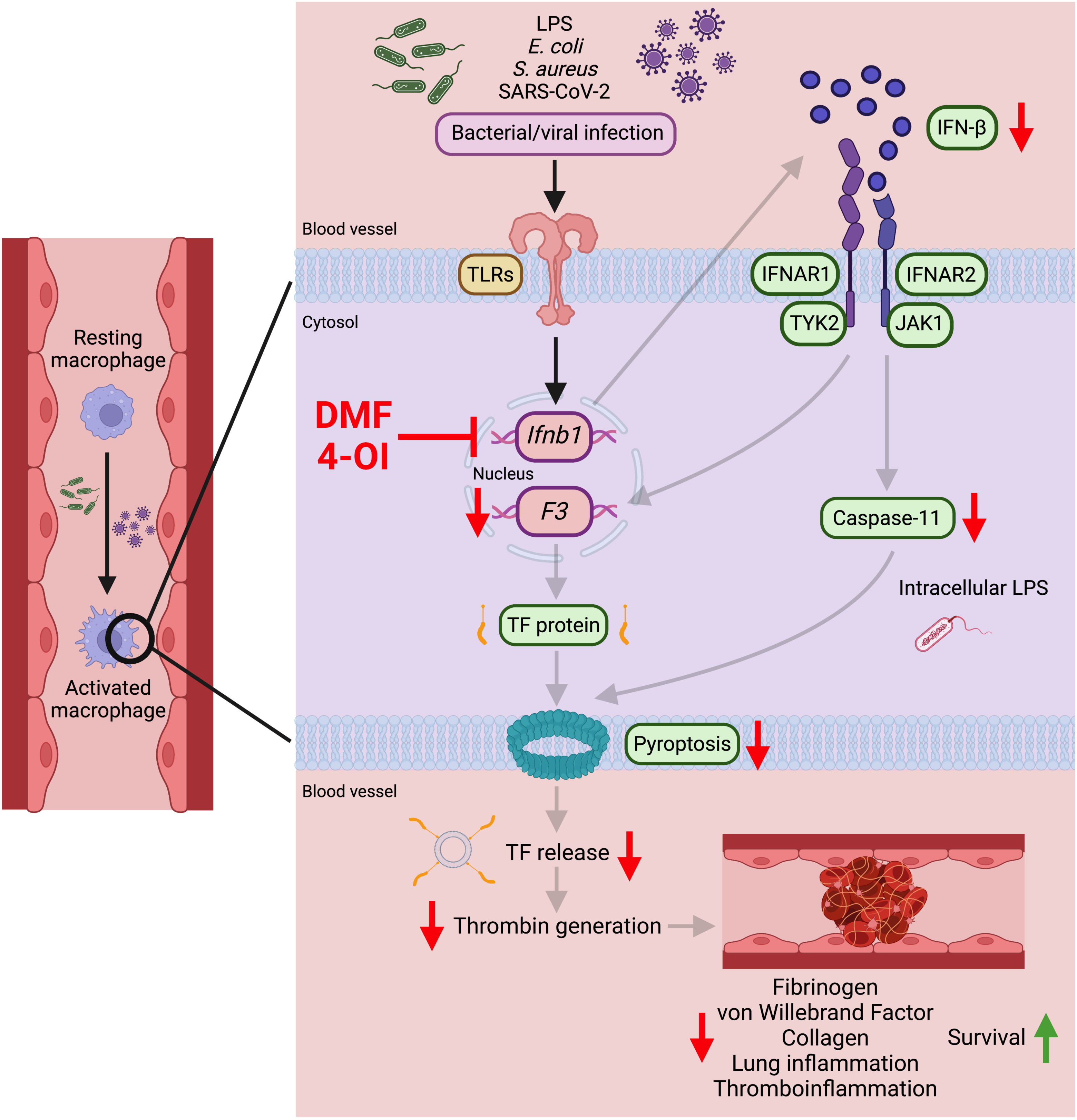
Resting macrophage
Activated macrophage
LPS
E. coli
S. aureus
SARS-CoV-2
Bacterial/viral infection
Blood vessel
TLRs
Cytosol
IFN-β
IFNAR1
IFNAR2
TYK2
JAK1
DMF
4-OI
Ifnb1
Nucleus
F3
Caspase-11
Intracellular LPS
TF protein
Pyroptosis
Blood vessel
TF release
Thrombin generation
Fibrinogen
von Willebrand Factor
Collagen
Lung inflammation
Thromboinflammation
Survival

132

Supplementary Figure 7 – DMF and 4-OI are anticoagulants that suppress inflammation-associated coagulation via inhibition of the macrophage type I IFN-TF axis

Schematic summarizing the anticoagulant effects of DMF and 4-OI. After bacterial or viral infection, *F3* is induced in macrophages via type I IFN signalling, as *F3* is a type I IFN-stimulated gene. This triggers excessive TF procoagulant activity and TF-dependent thrombin generation leading to coagulopathy. DMF and 4-OI block *F3* induction via inhibition of *Ifnb1*. DMF and 4-OI therefore suppress aberrant thrombin generation, downstream thromboinflammation, and improve survival in mice. Thus, the clinically approved drug DMF and anti-inflammatory tool compound 4-OI are anticoagulants that suppress inflammation-associated coagulation via inhibition of the macrophage type I IFN-TF axis.

Cell Line	Any				
Normal/Abnormal	Any				
Fold Change Up	2				
Fold Change Down	2				
Gene Name List	F3				
GeneName	Site	Core Match	Matrix Match	Start Site	End Site
F3					
	NFKB	1	0.988	64	76
	NFKB	1	0.998	60	76
	ISRE	1	0.963	797	812
	ICSBP	1	1	797	809
	IRF	1	0.995	793	804
	IRF	1	0.991	799	810
	NFKAPPAB65	1	0.991	65	75
	NFKAPPAB65	1	0.977	1387	1397
	IRF7	0.971	0.934	789	807
	IRF	1	0.996	795	810
	NFKB	1	0.972	63	77
	STAT3	1	0.925	737	753
	STAT3	0.99	0.981	872	888
	STAT3	0.974	0.973	875	891
	STAT1	0.912	0.896	871	892
	NFKAPPAB	0.986	0.992	65	75
	IRF8	1	1	798	805
	STAT3	1	0.826	1049	1070
	IRF	1	0.994	630	641
	IRF	1	0.99	636	647
	NFKAPPAB65	1	0.977	1354	1364
	IRF7	0.971	0.934	626	644
	IRF	1	0.986	632	647
	STAT1	1	0.999	1059	1067
	STAT1	1	0.856	1049	1070

Supplementary Table 1 – Predicted location of transcription factor sites in the region of the *F3* promoter

The coordinates of the predicted location of transcription factor sites in the region spanning -1500 bp to +500 bp from the start site of the *F3* promoter, generated using the publicly available Interferome database[42].

Source Data for Supplementary Figure 3a

JAK1 GAPDH

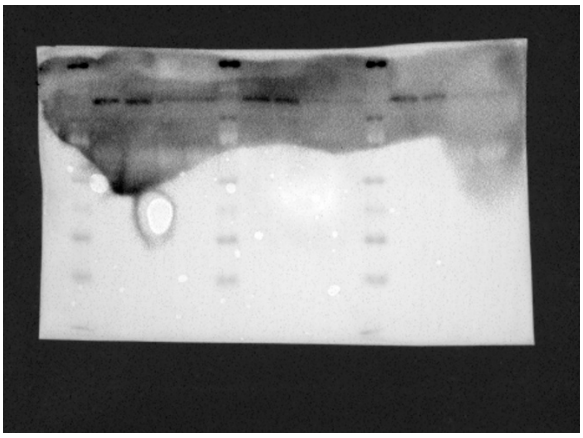

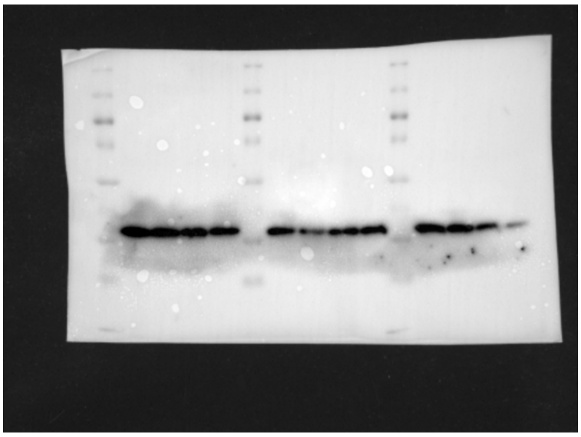

Supplement: Supplementary file 1 — Supplementary Information [file 41467_2023_39174_MOESM1_ESM.pdf]
