# Supplementary material for: Dimethyl fumarate and 4-octyl itaconate are anticoagulants that suppress Tissue Factor in macrophages via inhibition of Type I Interferon
Source: Nat Commun. 2023 Jun 14;14:3513. doi: 10.1038/s41467-023-39174-1 (PMC10265568; doi:10.1038/s41467-023-39174-1)
Supplement: Supplementary file 3 — Reporting Summary [file 41467_2023_39174_MOESM3_ESM.pdf]

nature portfolio

Corresponding author(s): Tristram A.J. Ryan, Luke A.J. O'Neill

Last updated by author(s): Apr 27, 2023

Reporting Summary

Nature Portfolio wishes to improve the reproducibility of the work that we publish. This form provides structure for consistency and transparency in reporting. For further information on Nature Portfolio policies, see our Editorial Policies and the Editorial Policy Checklist.

Statistics

For all statistical analyses, confirm that the following items are present in the figure legend, table legend, main text, or Methods section.

n/a	Confirmed	
☐	☒	The exact sample size (*n*) for each experimental group/condition, given as a discrete number and unit of measurement
☐	☒	A statement on whether measurements were taken from distinct samples or whether the same sample was measured repeatedly
☐	☒	The statistical test(s) used AND whether they are one- or two-sided<br>*Only common tests should be described solely by name; describe more complex techniques in the Methods section.*
☒	☐	A description of all covariates tested
☐	☒	A description of any assumptions or corrections, such as tests of normality and adjustment for multiple comparisons
☐	☒	A full description of the statistical parameters including central tendency (e.g. means) or other basic estimates (e.g. regression coefficient) AND variation (e.g. standard deviation) or associated estimates of uncertainty (e.g. confidence intervals)
☐	☒	For null hypothesis testing, the test statistic (e.g. *F*, *t*, *r*) with confidence intervals, effect sizes, degrees of freedom and *P* value noted<br>*Give P values as exact values whenever suitable.*
☒	☐	For Bayesian analysis, information on the choice of priors and Markov chain Monte Carlo settings
☒	☐	For hierarchical and complex designs, identification of the appropriate level for tests and full reporting of outcomes
☒	☐	Estimates of effect sizes (e.g. Cohen's *d*, Pearson's *r*), indicating how they were calculated

Our web collection on statistics for biologists contains articles on many of the points above.

Software and code

Policy information about availability of computer code

Data collection	Incucyte S3 Live-Cell Analysis System (Essen Bioscience), Thrombinoscope (Thrombinoscope BV), Leica Application Suite Advanced Fluorescence (Leica), Miramax slide scanner (Zeiss).
Data analysis	ImageJ 1.53t (NIH), GraphPad Prism 9.0, Image Lab Software 6.0.1 (Bio-Rad).

For manuscripts utilizing custom algorithms or software that are central to the research but not yet described in published literature, software must be made available to editors and reviewers. We strongly encourage code deposition in a community repository (e.g. GitHub). See the Nature Portfolio guidelines for submitting code & software for further information.

Data

Policy information about availability of data

All manuscripts must include a data availability statement. This statement should provide the following information, where applicable:

- Accession codes, unique identifiers, or web links for publicly available datasets
- A description of any restrictions on data availability
- For clinical datasets or third party data, please ensure that the statement adheres to our policy

RNA sequencing data (https://doi.org/10.5061/dryad.6wwpzgn28) is available via the Dryad Data Platform. Data generated from the Immunological Genome Project (ImmGen) bulk-population RNA-seq database, Interferome v2.0, and the ChIP-Atlas enrichment analysis tool are publicly available (and are their original publications are cited in the manuscript and Source Data files). Source data are provided with this paper in the Supplementary Information.

Field-specific reporting

Please select the one below that is the best fit for your research. If you are not sure, read the appropriate sections before making your selection.

- [x] Life sciences
- [ ] Behavioural & social sciences
- [ ] Ecological, evolutionary & environmental sciences

For a reference copy of the document with all sections, see nature.com/documents/nr-reporting-summary-flat.pdf

Life sciences study design

All studies must disclose on these points even when the disclosure is negative.

Sample size	At least 3 biological replicates were used for each in vitro experiment. Each in vitro experiment was performed on independent occasions unless otherwise stated. Each in vivo experiment was performed on at least 1 independent occasion. Although we did not use statistical methods to calculate sample size, we used a minimum of 3 biological replicates per experiment to account for biological variability, taking into account the 3 Rs principle and the fact that the majority of experiments were performed in primary murine macrophages from inbred mice. See statistical analysis section of methods for full details.
Data exclusions	No data were excluded from in vitro or in vivo experiments.
Replication	The in vitro and in vivo experiments were highly reproducible. Each in vitro experiment was repeated at least 3 independent times. The in vivo experiments were performed on 1-2 separate occasions per infection model, depending on time and availability of mice. In each case, in vivo experiments were successful and all data from mice included in the analysis to ensure reproducibility.
Randomization	For all in vitro experiments, primary bone marrow-derived macrophages were derived from each individual mouse (eg n=3 mice) and macrophages were counted and plated into eg. 12-well plates as necessary for each experiment. The wells were then labelled as per each treatment condition. Thus, as all cells from each mouse were pooled prior to plating, the cells in each experimental group were randomized. For all in vivo experiments, mice were age- and sex-matched and randomly assigned to treatment groups.
Blinding	The in vivo survival trial experiments were blinded to experimenters assessing and scoring mice clinically (see Fig. 3h and Supplemental Fig. 4b). All in vitro and the remaining in vivo experiments were not blinded due to lack of available experimenters with required expertise.

Reporting for specific materials, systems and methods

We require information from authors about some types of materials, experimental systems and methods used in many studies. Here, indicate whether each material, system or method listed is relevant to your study. If you are not sure if a list item applies to your research, read the appropriate section before selecting a response.

Materials & experimental systems

n/a	Involved in the study	
☐	☒	Antibodies
☒	☐	Eukaryotic cell lines
☒	☐	Palaeontology and archaeology
☐	☒	Animals and other organisms
☐	☒	Human research participants
☒	☐	Clinical data
☒	☐	Dual use research of concern

Methods

n/a	Involved in the study	
☒	☐	ChIP-seq
☒	☐	Flow cytometry
☒	☐	MRI-based neuroimaging

Antibodies

Antibodies used	Mouse-reactive anti-rat CASPASE-11 (14340), anti-rabbit GAPDH (2118), anti-rabbit JAK1 (3344), anti-rabbit alpha-TUBULIN (2144), and anti-rabbit TF (44861) were purchased from Cell Signaling and used for western blotting. Working dilutions of primary antibodies for western blotting were 1:1000. Horseradish peroxidase (HRP)-conjugated anti-rat (112-035-003) and anti-rabbit (111-035-003) IgG antibodies (both 1:2500) were purchased from Jackson Immunoresearch.<br><br>1H1 anti-TF antibody was a kind gift from Dr Helen Bettencourt (Genentech, Inc., South San Francisco, CA).
Validation	All western blotting antibodies have been validated for western blotting as listed in manufacturer's instructions. All antibodies have been validated for use in mice as per the manufacturer (Cell Signaling).<br><br>The 1H1 anti-TF antibody was generated and verified as an inhibitor of TF:FVIIa by Kirchhofer et al, 2005 (see citation 46 in the manuscript).

Animals and other organisms

Policy information about studies involving animals; ARRIVE guidelines recommended for reporting animal research

Laboratory animals	All experiments were performed with 6-12-week-old male and female mice on a C57BL/6JOlaHsd background unless stated below. Wild-type mice (WT) were bred in-house. Mice were kept in a 12-hour light/dark cycle and the facility was maintained at 20-24°C and 45-65% humidity. Caspase-11−/− mice on the C57BL/6J background were backcrossed onto the C57BL/6J background for another 8 generations. Heterozygous breeding pairs were used to generate WT and Caspase-11−/− littermates, and 6-12-week-old male and female mice were used for all relevant experiments. Ifnar-/- mice were also generated on the C57BL/6J background and age- and sex-matched WT male and female mice (6-12-weeks old) were used in all relevant experiments. In vivo models were performed with 6-12-week-old C57BL/6JOlaHsd mice and littermates were randomly assigned to experimental groups.
Wild animals	The study did not involve wild animals.
Field-collected samples	The study did not involve field-collected samples.
Ethics oversight	All mice were bred under specific pathogen-free conditions, under license and approval of the local animal research ethics committee (Health Products Regulatory Authority) and European Union regulations. All animal procedures were ethically approved by the Trinity College Dublin Animal Research Ethics Committee prior to experimentation, and conformed with the Directive 2010/63/EU of the European Parliament.

Note that full information on the approval of the study protocol must also be provided in the manuscript.

Human research participants

Policy information about studies involving human research participants

Population characteristics	For experiments performed in the School of Biochemistry and Immunology in TBSI (TCD) which involved isolation of human PBMCs for in vitro experiments, donors were defined as healthy and no other information was recorded. For experiments performed at the St James's, Tallaght University Hospital, Trinity Alliance for Research (STTAR) Bioresource, St. James's Hospital, Tallaght University Hospital, and Trinity Translational Medicine Institute (TCD) (for PBMCs isolated from SARS-CoV-2-infected patients), blood samples were obtained anonymously and written informed consent for the use of blood for research purposes was obtained from the donors. No other information was recorded. There were no significant covariates between the groups.
Recruitment	For all experiments using human samples (performed in the School of Biochemistry and Immunology in TBSI (TCD), St James's, Tallaght University Hospital, Trinity Alliance for Research (STTAR) Bioresource, St. James's Hospital, Tallaght University Hospital, and Trinity Translational Medicine Institute (TCD)), all participants provided samples anonymously and informed written consent for the use of blood for research purposes. Healthy controls and SARS-CoV-2-infected patients voluntarily donated blood. No selection bias was noted.
Ethics oversight	School of Biochemistry and Immunology Research Ethics Committee (TCD); National Research Ethics Committee (STTAR); TCD guide on good research practice, which follows the guidelines detailed in the National Institutes of Health Belmont Report (1978) and the Declaration of Helsinki.

Note that full information on the approval of the study protocol must also be provided in the manuscript.
